# Diversity of HBV genotypes and their association with precore/basal core mutations among HBsAg-positive patients in Ibadan, Nigeria

**DOI:** 10.1099/acmi.0.000821.v3

**Published:** 2024-11-07

**Authors:** Adedayo Omotayo Faneye, Babatunde Olanrewaju Motayo, Aisha Mustafa, Georgina Odiabo

**Affiliations:** 1 Department of Virology, College of Medicine, University of Ibadan, Ibadan, Nigeria; 2 Department of Medical Microbiology, Federal Medical Centre, Abeokuta, Nigeria

**Keywords:** hepatitis B virus, molecular characterization, mutational profile, Nigeria

## Abstract

**Background.** Hepatitis B virus (HBV) is the most implicated cause of severe liver disease and hepatocellular carcinoma worldwide. Studies have shown that the basal core protein (BCP) and precore protein (PC) of HBV play a significant role in HBV-related carcinogenesis. There is a paucity of data on the type and effect of BCP and PC mutations in Nigeria. This study aims to genotype HBV and investigate any mutations within the BCP and PC among HBV patients in Ibadan, Nigeria.

**Methods.** Forty HBV-DNA-positive patients were recruited into this study, and the viral load assay and genotyping by nested multiplex PCR were done. The partial X gene region was amplified and Sanger sequenced. The BPC and PC genomic regions were then analysed using bioinformatics.

**Results.** Twenty-three participants recorded HBV DNA viral load of >20 000 IU, while 17 had <20 000 IU and 28 samples were genotyped. Five genotypes (A, B, C, D and E) and four mixed genotypes (AC, AD ACD and ABCD) were detected. Genotype AC was the most frequently encountered, while genotypes E and B were the least encountered. Mutation was highest in ages 34–45 years. Double mutation A1762T and G1764A within the BCP region was the most encountered mutation.

**Conclusions.** We report a diverse HBV genetic landscape, with mixed infections between genotypes with BCP double-mutation A1762T/G1764A, signalling the likelihood of poor HBV-related liver disease prognosis. Our findings contribute to our understanding of the molecular characteristics of HBV and its potential implications for disease progression and management among HBV-infected Nigerians.

## Data summary

The sequence data in this study were generated by Sanger sequencing, and the sequence data have been archived and are freely available on GenBank under the accession numbers PP412003 and PP436740–PP436746. Supplementary materials can be found in Figshare doi: https://doi.org/10.6084/m9.figshare.26863414.v1 [1].

## Introduction

Hepatitis B (HB) is a global health problem and the major causative agent for viral hepatitis. Hepatitis B virus (HBV) is an enveloped partially double-stranded DNA virus belonging to the family Hepadnaviridae and genus *Orthohepadnavirus* [2]. HB viral infection has the potential to result in persistent long-lasting infection, resulting in chronic illnesses such as chronic hepatitis B (CHB) infection, liver cirrhosis and hepatocellular carcinoma (HCC) [3]. The World Health Organization estimates that 296 million people were living with CHB infection in 2019, with 1.5 million new infections each year. Also, HB resulted in an estimated 820 000 deaths, mostly from cirrhosis and HCC [4].

HBV is one of the most genetically diverse DNA viruses. It has a genome consisting of four distinct ORFs; the presurface antigen/surface antigen gene (preS/S), the precore/core gene (pre-C/C), the polymerase gene (P) and the X gene (X). Due to the absence of proofreading activity during reverse transcription and its rapid replication rate, HBV naturally generates quasispecies, which often emerge in response to antiviral treatments [5]. The virus is classified into ten genotypes (A to J), each with its own geographical distribution [6]. During the course of chronic HBV infection, the transition from hepatitis B e antigen (HBeAg), to its antibody (anti-HBe) is typically associated with a decline in HBV replication and the resolution of hepatitis [7]. Therefore, HBeAg seroconversion is considered beneficial for patients with CHB. However, there are cases where individuals continue to have high levels of ongoing liver disease despite seroconversion [8,9]. Mutations occurring within the precore (pre-C) region of the HBV genome have been frequently reported to be responsible for the development of an HBeAg-negative profile [10]. These mutations result in the downregulation of the pre-C and core genes, causing low level to zero transcription, leading to the absence of HBeAg secretion [10,11]. The core promoter region plays a crucial role in viral replication, morphogenesis and pathogenesis [12,14]. Similarly, the precore region also contributes significantly to viral replication. Mutations in both the core promoter and precore regions allow the virus to evade host immune surveillance, resulting in the development of mutated strains that may exhibit altered pathogenicity [15]. There is a growing global concern about HBeAg-negative CHB infections becoming the most frequent type of HBV. Genotypes and mutations of HBV are important viral biomarkers in the prognosis of disease progression and also assist in the diagnosis to help suggest the type of therapy that can be used [16,19]. In Nigeria, there is a high burden of HBV infection. Analysis of the correlation between the high viral titre and genotype/precore/basal core promoter variations in HBV patients holds contextual significance. It will provide valuable data and insights contributing to a better understanding of local HBV epidemiology, genotype distribution and disease outcomes in the Nigerian population.

The need to fill in this gap in knowledge conceptualised this study, which aims to identify HBV genotypes and the precore and basal core promoter mutations in patients with high viral load in Ibadan, Nigeria.

## Methods

### Study design and population

**Fig. 1.**
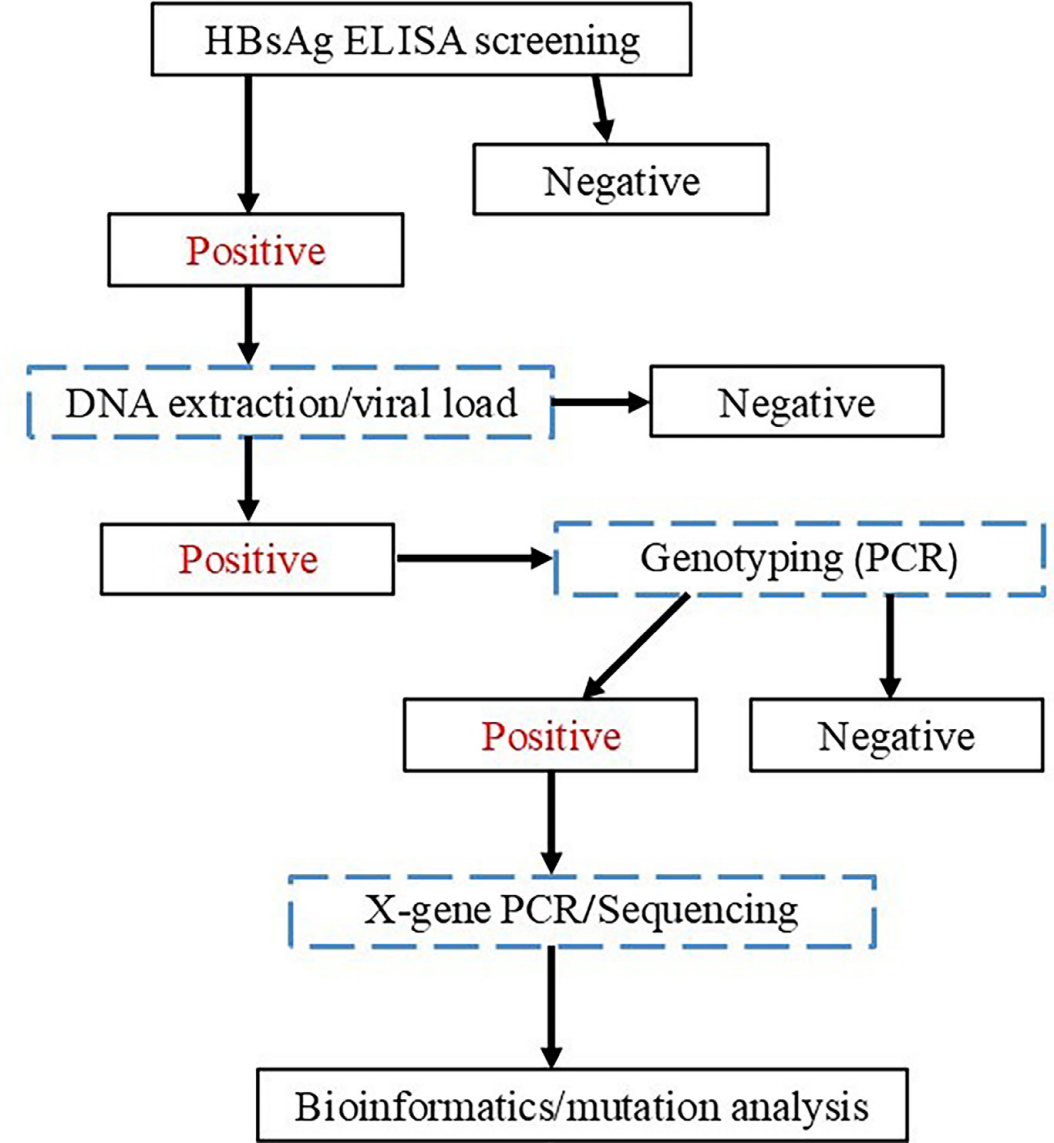
Flow chart showing the summary of study design and laboratory strategy for the genotyping of HBV and precure/core gene sequencing and characterisation among hepatology patients attending UCH, Ibadan, Nigeria.

The current study is a cross-sectional study carried out between May 2023 and September 2023 to determine the relationship between viral titre, genotype precore and basal core promoter mutation among HBV-infected patients in university college hospital (UCH), Nigeria. The study was carried out at the Department of Virology, University College Hospital, Ibadan, Oyo State, Nigeria. Ibadan city is the most populous city in Oyo State, Nigeria, with a population of over 3 million people of majorly Yoruba ethnicity. A flow chart showing the summary of the study design and protocol is shown in Fig. 1. Ethical approval was sought and given with the number UI/UCH-REC. 19-002; participants also gave written informed consent before enrolment into the study. Five millilitres of whole blood was collected by venipuncture from consenting participants into EDTA bottles, and the plasma fraction was separated by centrifugation from the sample before further testing.

### DNA extraction and viral load assay

Genomic DNA was extracted from each of the samples using Zymo Direct-zol miniprep extraction kit, Zymo research, Irvine, CA, USA. Briefly, 50 μl of proteinase K was added to a sterile centrifuge tube, 200 µl of sample was added to it, 200 µl of lysis buffer was added and the mixture was vortexed for 15 s and centrifuged for 10 s. It was then pre-heated for 10 min at 72 °C using the heating block.

About 250 µl of absolute ethanol was added to the mixture and vortexed for 15 s before transferring into a labelled spin column. The mixture was centrifuged at 120 00 ***g*** for 1 min and the spin column was placed into a new collection tube; 500 µl inhibitor remover was added to the spin column and then centrifuged at 120 00 ***g*** for 1 min. The spin column was then washed twice with 500 µl of deionised wash solution and centrifuged.

The spin column and a fresh collection tube were centrifuged for 3 min to remove residual ethanol. The spin column was then fit into a new 1.5 ml centrifuge tube, the cover of the spin column was opened and it was placed in the heating block at 72 °C for 2 min. Thereafter, 50 µl of eluent buffer was added to the spin column and centrifuged to elute the nucleic acid from the spin column. The resultant extracted DNA sample was preserved at a temperature of −20 °C.

Viral DNA was assayed for HBV viral load by real-time PCR using a commercial kit by Elizabeth Pharmacon (Czech Republic) on an ABI 7500 fast real-time thermocycler (Applied Biosystems, Foster City, California, USA).

### Genotyping by nested PCR

The detection and genotyping of HBV DNA were performed using nested PCR in this study. Multiplex-nested PCR was employed with type-specific primers [20], as shown in Table S1 (available in the online version of this article). Positive control isolate (MK673283) was used as standard HBV control for the assays. The amplification protocol followed the protocol proposed by Naito *et al*. [20].

### Basal core protein/basal core protein amplification and sequencing

With a brief modification of PCR condition and reaction mixture, the basal core protein (BCP)/precore protein (PC) genome regions from the EcoRI site (nt 1653 ± 1959) were amplified using a set of primers, which were used as in Belyhun *et al*. [21], and show in Table S2. The BCP/PC nt codons covered the partial X protein, full precore and partial core protein (C) genome regions. Amplification conditions were as previously reported [21]. Amplicons generated from the BCP/PC PCR reactions were purified with a purification kit (Jena Bioscience, Hilden, Germany). Purified amplicons were sequenced by Sanger sequencing using a big dye chemistry kit on an ABI 3500 genetic analyser (Applied Biosystems) by a commercial laboratory. The HBV partial X gene sequences generated from this study were submitted to GenBank under the accession numbers PP412003 and PP436740–PP436746.

### Bioinformatic analysis

The HBV pre-core/core gene sequences were subjected to the hepatitis virus database identification of the HBV so as to ensure that the sequences obtained matched only HBV sequences. A total of four reference sequences of HBV genotypes A, D and E with a universal reference for HBV were obtained from the NCBI database and were used for comparison with the sequences of the isolates in this study (genotype A: X70185, genotype D: X72702 and genotype E: X75657). The sequences and all the reference sequences were aligned using MAFFT software. The phylogenic analysis was done using IOTree [22] with the GTR+I model [23]. The HBV pre-core/core gene sequences were re-aligned according to their genotypes along with genotype-specific reference sequences. Sequence variation and detection of mutations affecting HBeAg expression at the BCP transcriptional (BCP double mutations; A1762T/G1764A) and translational genes (Kozak sequences mutants, nt1809–1812) and PC initiation (1814±1816), translational stop codon (G1896A with C1858T) and post-translational mutant gene (G1862T) were all noted and identified.

The sequences were subsequently translated into amino acid residues, and the amino acid variations were also noted.

### Statistical analysis

Data obtained were analysed using the Rstudio (www.rporject.comhttps://www.r-project.org/). Analyses were carried out using descriptive statistics with chi-square test and Fisher’s test.

## Results

**Fig. 2.**
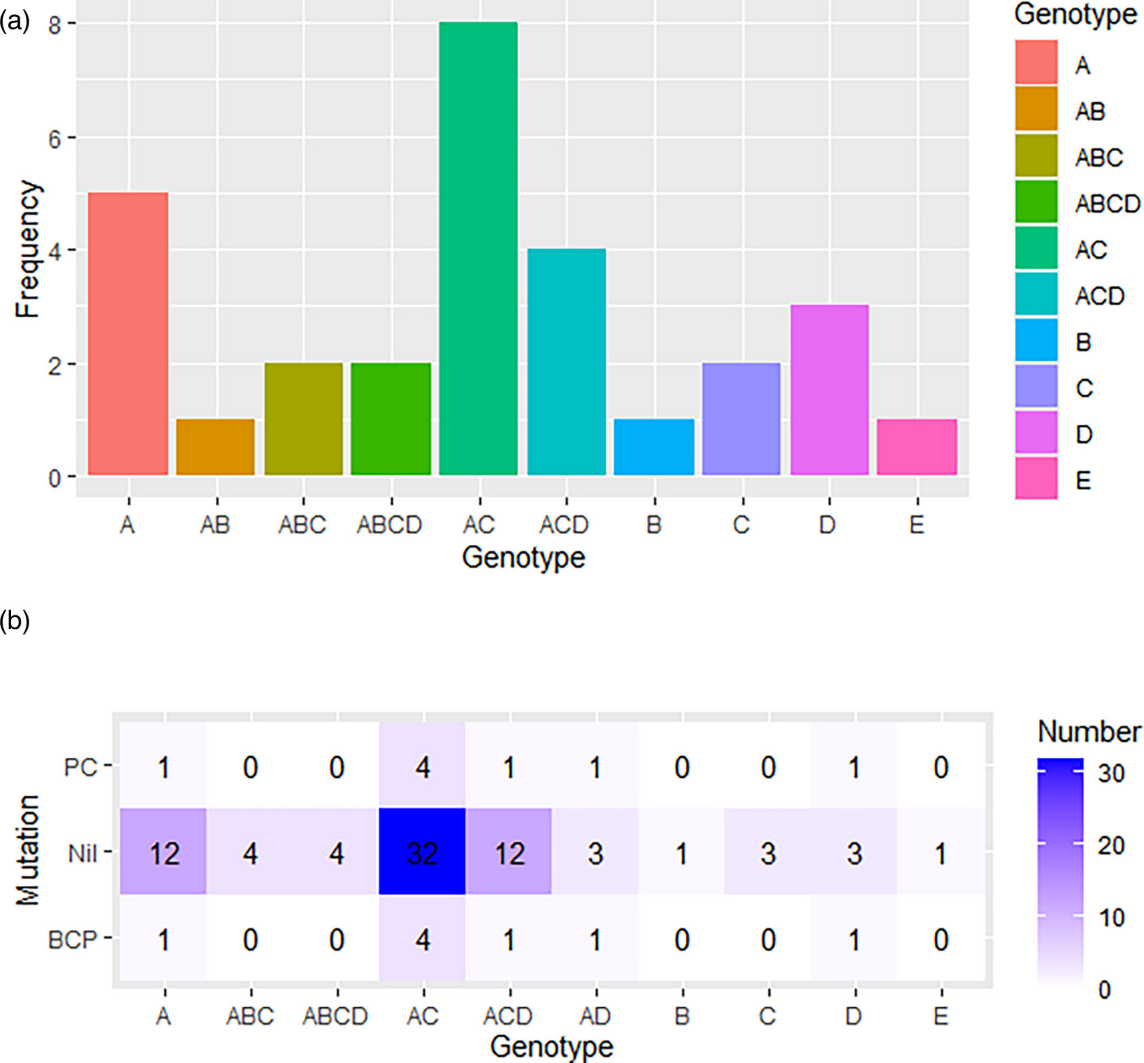
(a) Bar chart showing distribution frequency of HBV genotypes among the HBV-infected individuals in Ibadan, Nigeria. (b) Heat map showing mutation occurrence according to the genotype among HBV-infected persons in Ibadan, Nigeria.

Forty HBsAg-positive samples with detectable HBV DNA were selected based on viral copy number (viral load). The samples were drawn from a pool of HBsAg-positive individuals screened during hospital visits to the gastroenterology clinic at the UCH in Ibadan. A total of 23 individuals recorded HBV DNA viral load of >20 000 IU, while 17 had a viral load of <20  000 IU; 28 samples out of 40 were successfully genotyped by snPCR; agarose gel picture of an snPCR run showing positive samples is shown in Fig. S1. Results from genotyping gave five genotypes A, B, C, D and E, with an array of mixed-genotype infections. The mixed infection with genotype constellation of AC was the most abundantly identified genotype, while genotypes B and E were the least encountered genotypes (Fig. 2a). Frequency of BCP/PC mutations according to the genotype shows that the genotype AC had the highest number of sequences recording mutations relative to the WT sequence analysed, while genotypes A, AD, ACD and D recorded only one sequence with mutation (Fig. 2b).

**Fig. 3.**
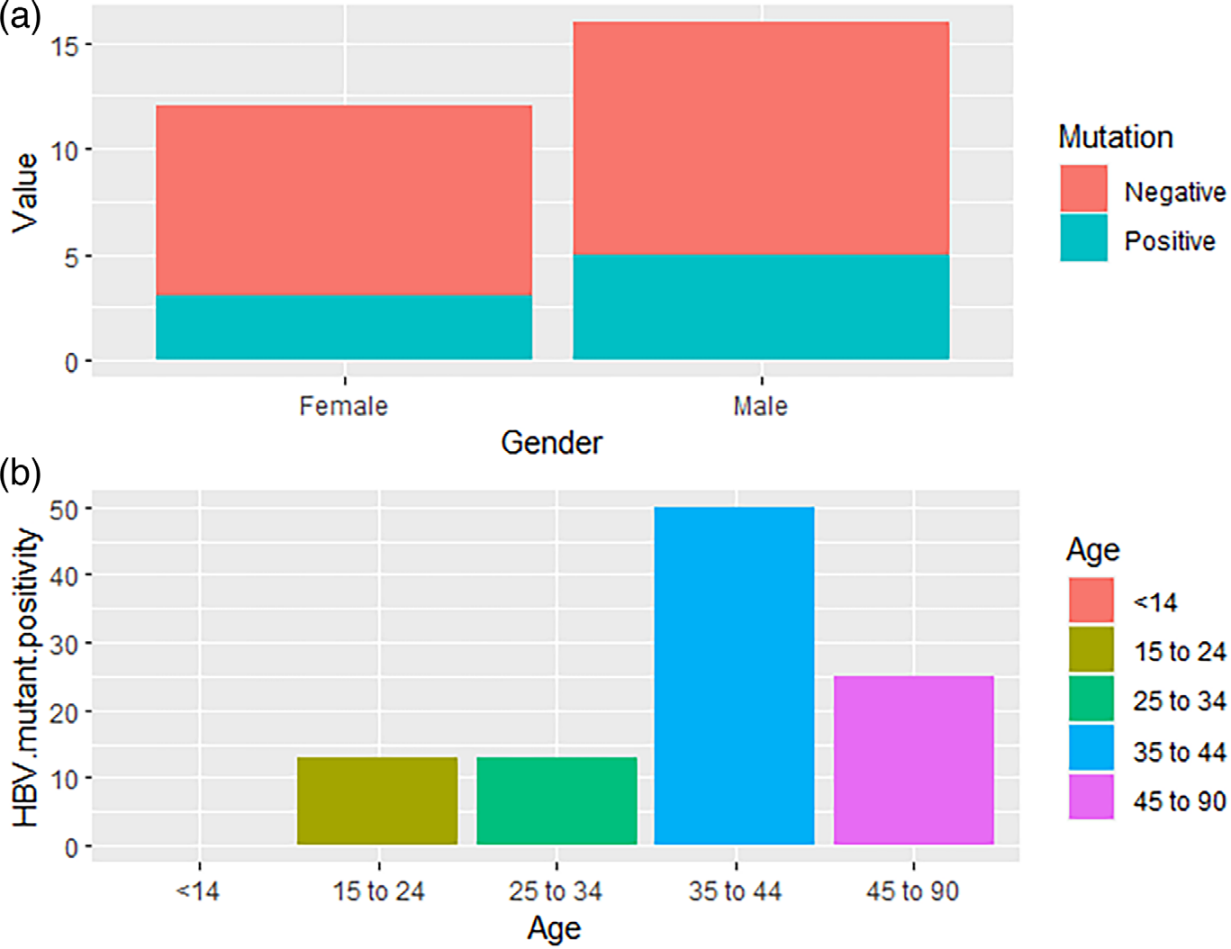
(a) Bar chart representing the number of mutated isolates according to gender; (b) Bar chart representing the percentage distribution of mutant-positive viruses according to the age range.

**Fig. 4.**
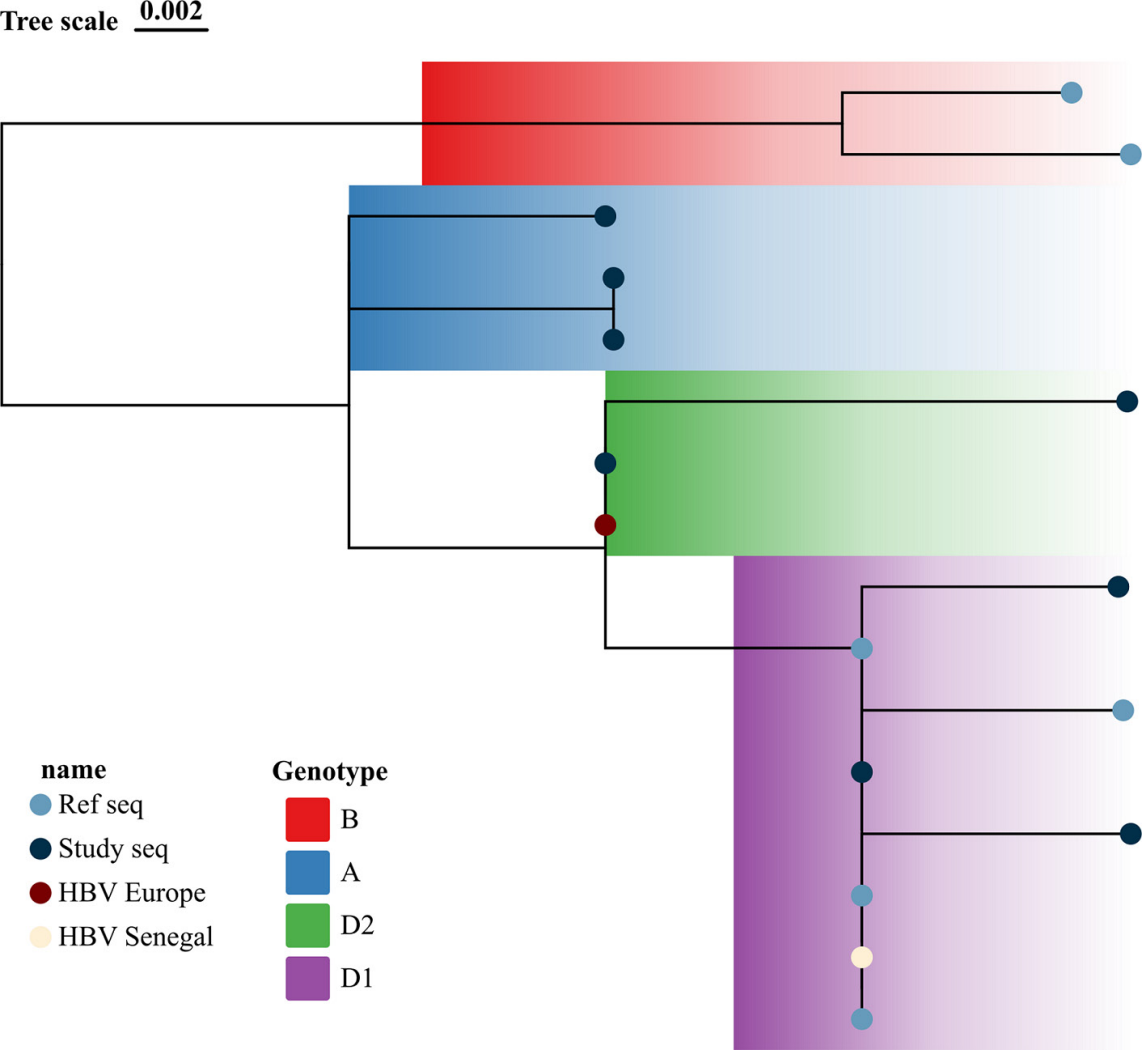
Maximum-likelihood phylogenetic tree of the partial X gene of HBV isolated from individuals in UCH Ibadan, Nigeria, along with reference HBV sequences. Reference sequences include HBV genotype D1 reference accession no: JF754635; genotype D2 reference accession no: X72702.

**Fig. 5.**
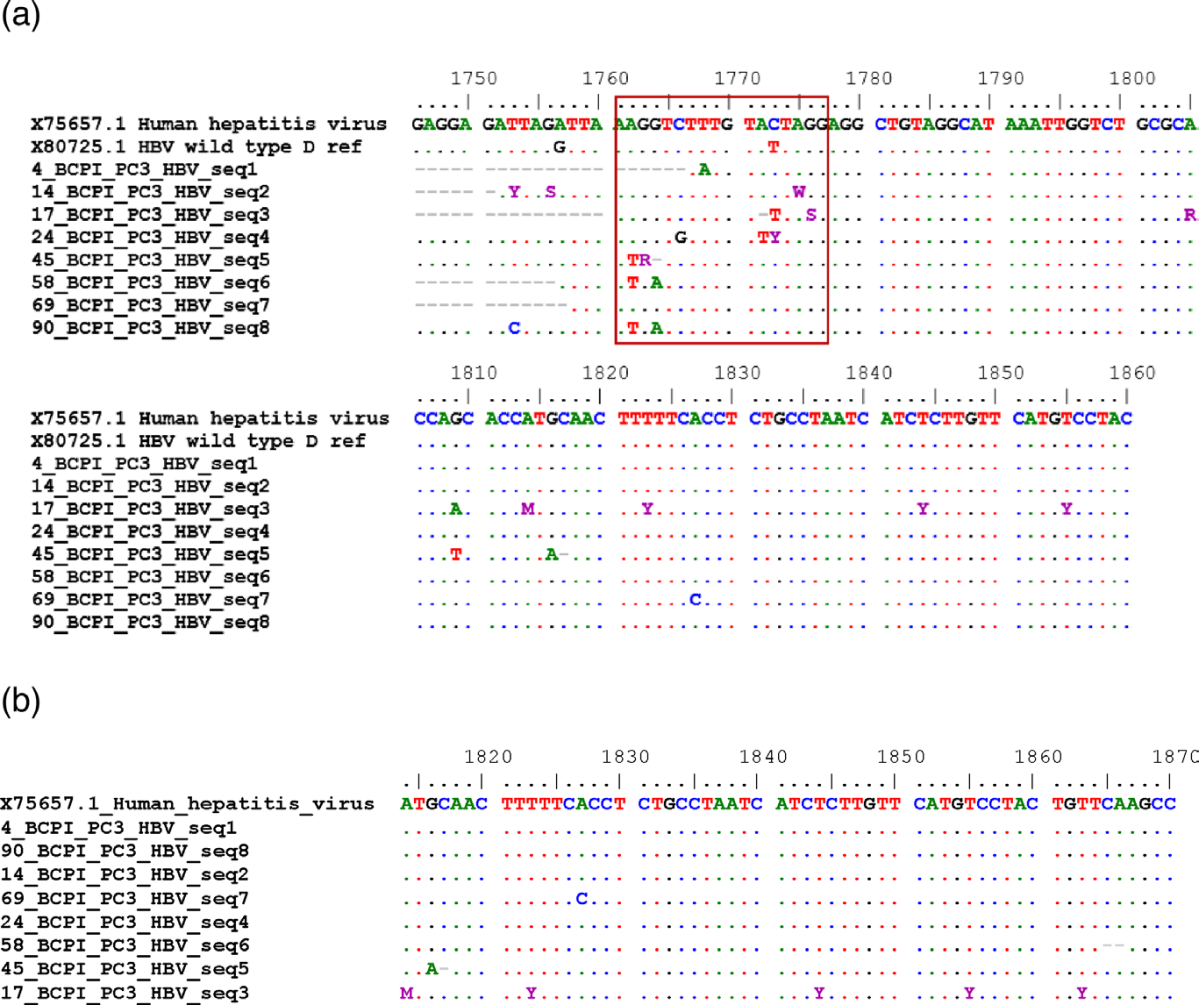
(a) Nucleotide sequence alignment of the partial BCP region of HBV (nt1746–1860) from Ibadan, with reference to WT genotype A and D; the transparent red box represents the region of major substitutions. (b) Nucleotide sequence alignment of the partial PC region (nt1814–1870), with reference to genotype A; the arrows show locations of substitutions.

Fig. 3a shows the BCP/PC mutant distribution according to gender with males having a higher mutation frequency than females. Mutant distribution according to age range shows that most affected ages are between 35 and 44 years with 50% mutation rate, while ages 45–90 recorded 25% and the other age groups 12.5% (Fig. 3b). Nucleotide sequence alignment and amino acid sequence alignments of the study isolates along with reference HBV reference isolate GenBank accession no X70185 are shown in Fig. S2. The phylogenetic analysis of the partial X gene of our study isolates revealed that the majority of the study sequences clustered within the subgenotype D1, while two sequences clustered within D2 and three other sequences clustered in genotype A, as shown in Fig. 4. Nucleotide sequence alignment of the BCP region nt1742 to 1849 is represented in Fig. 5a, with the major mutagenic region in the red box, while the Kozak mutant region is shown in the sky blue shaded box (nt1809 to 1812). The PC alignment is also shown in Fig. 5b relative to reference WT genotype A (Accession no X70185). Table 1 shows the nt mutations relative to the reference sequences.

**Table 1.** Mutations within the BCP/PC gene region of HBV-positive samples from Ibadan

Isolate	Genotype	BCP mutation (1742−1849)	PC mutation (1814–1904)
**4**	AD	T1768-A	
**14**	AC	T1753-Y, G1756-S, A1775-W	
**17**	AC	C1773-T, G1776-S, A1805-R, G1809-A, A1814-M	A1814-M
**24**	AC	C1766-G, A1772-T, C1773-Y	
**45**	ACD	A1762-T, A1763-R, G1808-T, G1806-A	G1816-A
**58**	D	A1762-T, G1764-A	
**69**	AC	C1826-A	
**90**	A	C1753-A, A1762-T, G1764-A	A1827-C, T1823-Y, T1844-Y, T1855-Y, T1863-Y

## Discussion

In this study, we investigated BCP and PC mutations in relation to HBV genotypes and some demographic (age and gender) features in patients chronically infected with HBV from the UCH Ibadan, Nigeria. Viral titres of all the samples in this study exceeded 2000 IU ml^−1^, indicating a high viral load among the study population, with 31.1% having a viral load greater than 20  000 IU ml^−1^. This indicates a high viral titre of the participants with a significant proportion at a greater risk of liver damage and cirrhosis [24,25]. Genotype A was found to be the most prevalent, accounting for 15.7% of the cases, while genotypes B and E were the least common, each representing only 1.2% of the cases. The presence of mixed infections involving genotypes A, B, C and D further highlights the genetic diversity of HBV in the studied population. Notably, the combination of genotypes A and C (AC) exhibited the highest prevalence at 40%; this is in agreement with several studies that have reported a high rate of mixed-genotype infections [26,28]. Our findings seemed to be consistent with previous studies, which reported a higher prevalence of mixed-genotype infections compared to single-genotype infections 58.3 and 41.7% [29,30]. We reported genotype A as the most prevalent single genotype, which is in contrast to some reports in other studies conducted in Nigeria and other West African countries, where genotype E was more prevalent [31,33].

The prevalence of PC/BCP mutations in relation to age revealed that ages 35–44 exhibited the highest prevalence (50%) of BCP and PC mutations, suggesting a potential age-dependent association with these mutations. In addition, males demonstrated a higher prevalence (53.3%) compared to females (47.5%). These observations raise interesting questions regarding the role of age and gender in HBV evolution and mutagenesis; further studies are required to explore these associations in greater depth. The distribution of PC/BCP mutations in relation to genotypes revealed that mixed AC genotype displayed the highest prevalence (50%), indicating a potential interaction or synergistic effect between genotypes A and C in HBV evolution and mutagenesis. A major limitation was our inability to resolve which genotype was responsible for which mutation among the mixed genotype samples, although only genotypes AC and AD were positive for BC/BPC mutants. This is an indication of the strong involvement of genotype A in HBV mutagenesis, as genotype A was the most frequently mutated among singly genotyped samples. Phylogenetic analysis of the partial X gene shows that the majority of the study sequences clustered within genotype D with only two sequences clustering within genotype A, as shown in Fig. 4.

The BCP mutations of A1762T and G1764A were found within the basal core region with a frequency of 4.4% each. The mutation A1762T represents a change in amino acid lysine to asparagine, while G1764A is from glycine to aspartic acid. The double baal core mutation observed (A1762T/G1764A) has been associated with HBeAg negativity and advanced liver disease [14,34]. Liu *et al*. [35] reported that the BCP double mutation A1762T/G1764A was associated with the progression of HCC, independent of HBV genotype and viral load. In this study, genotype A had the highest double mutation rate. A study from North India reported conversely that genotype D had a higher frequency of BCP mutations A1762T/G1764A compared to patients infected with genotype A [36]. Another BCP mutation identified in our study was T1753C, with a frequency of 3.3%; however, there was no amino acid change. A triple mutation of A1762T/G1764A and T1753V was found in the sample with the T1753C mutation, while another sample also displayed a similar profile. A study from East Kalimantan, Indonesia, linked this triple mutation to an increased risk of advanced liver disease [37]. The T1753C mutation has been recognised as a hotspot mutation in the protein-X encoding gene, shown to enhance the transactivation and antiproliferation activity of protein-X in HBV genotype D, thereby contributing to carcinogenesis [38]. In our study, we observed the G1816A mutation in genotype A; this also corresponds to a study from Kenya, which reported that mutations in the Kozak region (1809–1812) or precore start codon (1814–1816) were responsible for HBeAg negativity in patients with subgenotype A1 [39]. The main limitations of this study include the small number of samples sequenced, and the lack of sufficient clinical data from the patients at the time of enrolment to allow for proper clinical profiling.

## Conclusion

This study provides insights into the distribution of BCP and PC mutations in relation to HBV genotypes and their associations with demographic and clinical characteristics. Our study provides insights into the prevalence and significance of specific BCP and precore/core mutations in HBV-infected patients in Nigeria. The findings of this study have shown that HBV-infected Nigerians are at a higher risk of poor clinical prognosis and severe liver disease and add to our understanding of HBV-related liver disease in Nigeria. We therefore recommend that larger studies be conducted across different regions of Nigeria to provide a more comprehensive understanding of the distribution and prevalence of HBV genotypes and their association with different mutations and clinical outcomes.

## supplementary material

10.1099/acmi.0.000821.v3 Uncited Fig. S1.

10.1099/acmi.0.000821.v3 Uncited Fig. S2.

10.1099/acmi.0.000821.v3 Uncited Table S1.

10.1099/acmi.0.000821.v3 Uncited Table S2.

## Abbreviations

BCP: basal core protein
CHB: chronic hepatitis B
HBV: hepatitis B virus
HCC: hepatocellular carcinoma
PC: precore protein
