# Supplementary material for: Diversity of HBV genotypes and their association with precore/basal core mutations among HBsAg-positive patients in Ibadan, Nigeria
Source: Access Microbiol. 2024 Nov 7;6(11):000821.v3. doi: 10.1099/acmi.0.000821.v3 (PMC11542583; doi:10.1099/acmi.0.000821.v3)
Supplement: Uncited Fig. S1. [file acmi-6-00821-s001.pdf]

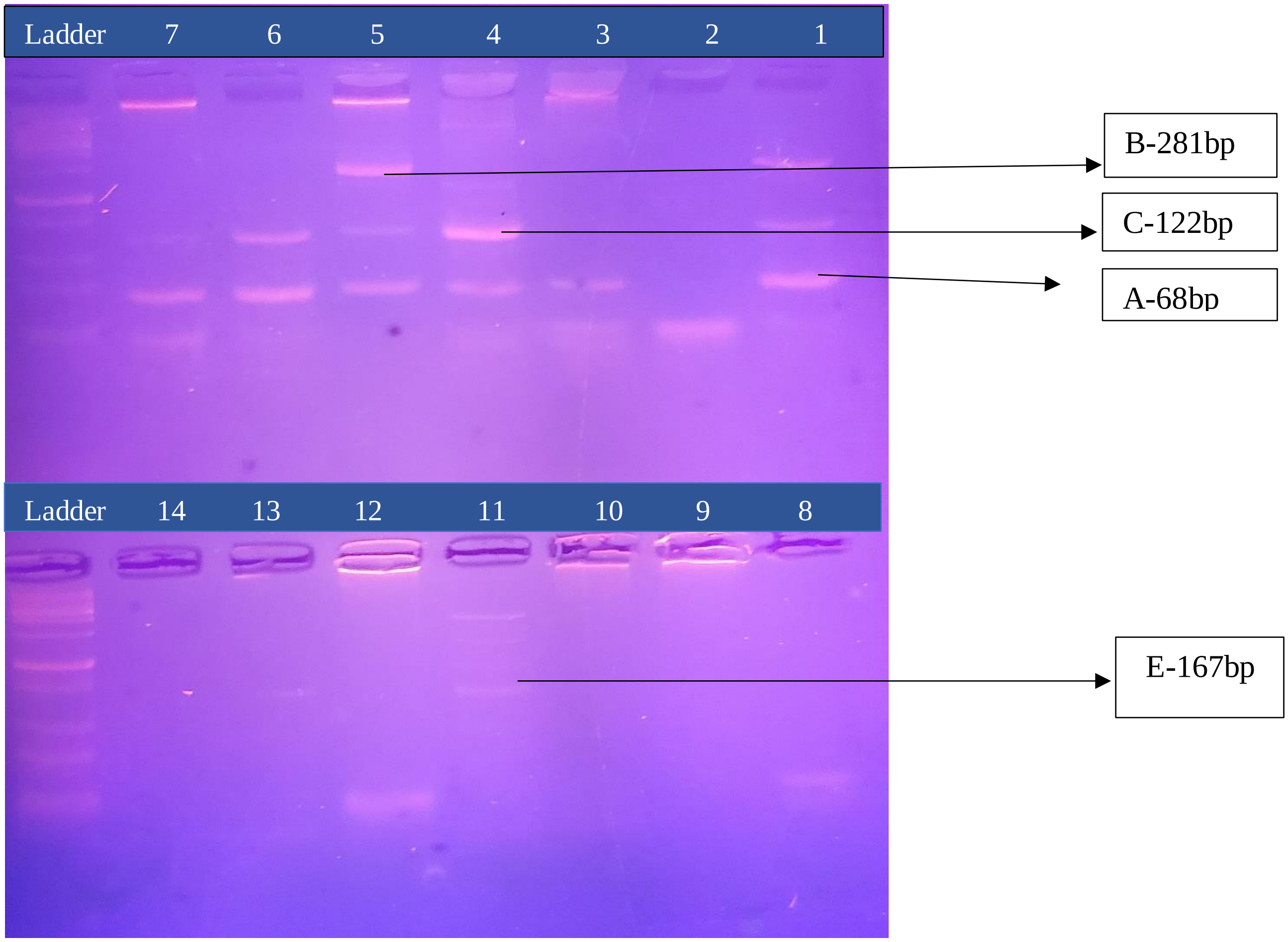

Supplementary figure. 1 Agarose Gel Electrophoresis Image Showing Amplified Products of the genotypes of HBV

Band Size: A- 68bp, B-281bp, C-122bp, E-167bp.

Legend: Lane 3-14 are samples
