# Supplementary figures and images for: Diversity of HBV genotypes and their association with precore/basal core mutations among HBsAg-positive patients in Ibadan, Nigeria
Source: Access Microbiol. 2024 Nov 7;6(11):000821.v3. doi: 10.1099/acmi.0.000821.v3 (PMC11542583; doi:10.1099/acmi.0.000821.v3)

## A

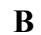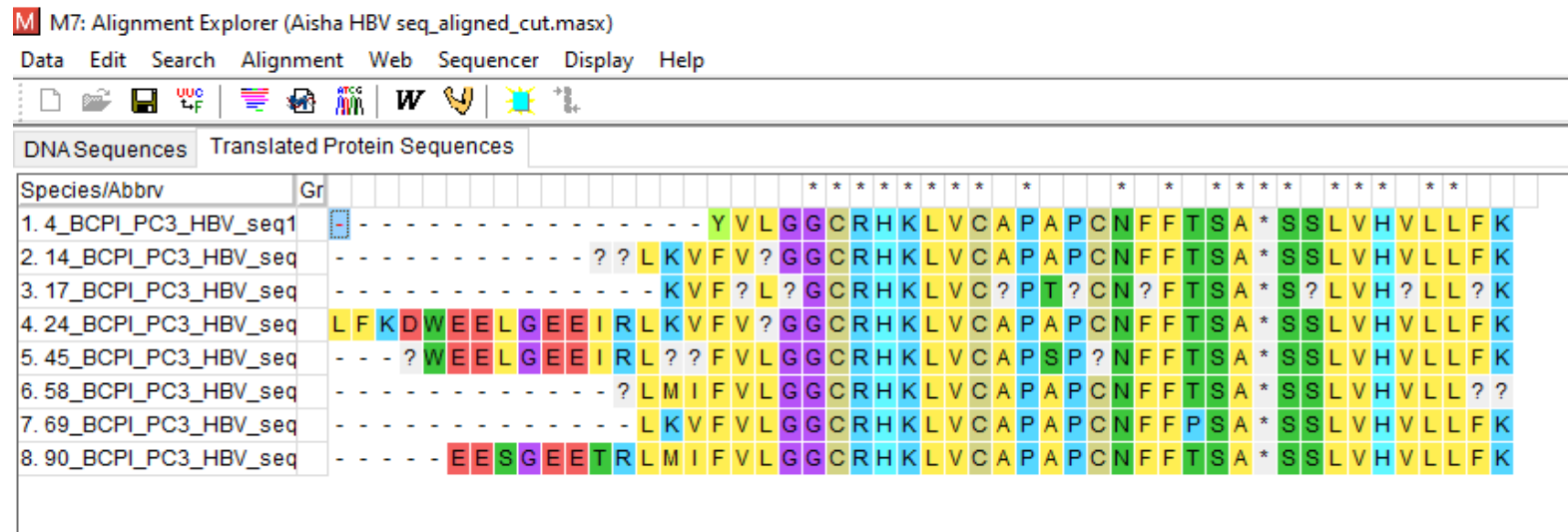

Supplement: Uncited Fig. S2. [file acmi-6-00821-s002.pdf]
