# Supplementary material for: Diversity of HBV genotypes and their association with precore/basal core mutations among HBsAg-positive patients in Ibadan, Nigeria
Source: Access Microbiol. 2024 Nov 7;6(11):000821.v3. doi: 10.1099/acmi.0.000821.v3 (PMC11542583; doi:10.1099/acmi.0.000821.v3)
Supplement: Uncited Table S1. [file acmi-6-00821-s003.pdf]

SUPPLIMENTARY TABLE 1: Sequences of primers for hepatitis B virus amplification and genotyping used by multiplex-nested polymerase chain reaction

**Primer**	**Sequence (5'-3')**	**Specificity**	**Position**	**Polarity**	**Gene location**	**Amplicon size**
1st round PCR						
P1	TCACCATATTCTTGGGAACAAGA	Universal	2823-2845	Sense		
S1-2	CGAACCACTGAACAAATGGC	Universal	685-704	Antisense	S1/S2	
2nd round PCR						
B2	GGCTCCAGTTCCGGAACAGT	Type A-E	67-86	Sense		
BA1R	CTCGCGGAGATTGACGAGATGT	Type A	113-134	Antisense	S1	68bp
BB1R	GGTCCTAGGAATCCTGATGTTG	Type B	165-186	Antisense	S1	281bp
BC1R	CAGGTTGGTGAGCTGGAGA	Type C	2979-2996	Antisense	S1	122bp
2nd round PCR		-				
Mix B						
B2R	GGAGGCGGATTTGCTGGCAA	Type D-F	3078-3097	Antisense		
BD1	GCCAACAAGGTAGGAGCT	Type D	2979-2996	Sense	S2	120bp
BE1	CACCAGAAATCCAGATTGGGACCA	Type E	2955-2978	Sense	S2	167bp
BF1	GTTACGGTCCAGGGTTACCA	Type F	3032-3051	Sense	S2	97bp
