# Supplementary material for: Diversity of HBV genotypes and their association with precore/basal core mutations among HBsAg-positive patients in Ibadan, Nigeria
Source: Access Microbiol. 2024 Nov 7;6(11):000821.v3. doi: 10.1099/acmi.0.000821.v3 (PMC11542583; doi:10.1099/acmi.0.000821.v3)
Supplement: Uncited Table S2. [file acmi-6-00821-s004.pdf]

Supplementary Table 2. Primer sequence for BCP/PC amplification

Primer	Sequence (5’-3’)	Position	Polarity	Amplicon size
1ST round PCR				
BCP/PC 1	5’-GCATGGA GACCACCGTGAAC-3’	1606±1625	Sense	330
BCP/PC 2	5’-GGAAAGAAGTCCGAGGGCAA-3’	1974±1955	Anti-sense	
2nd round PCR				
BCP/PC 3	5’ CATAAGAGGACTCTTGGACT-3’	1653±1672	Sense	
BCP/PC 4	5’GGCAAAAAACAGAGTAACTC-3’	1959±1940	Anti-sense	268
